# Ultrasonographic assessment of the diaphragm in systemic lupus erythematosus: cut-off values and predictive indicators of diaphragm thickness

**DOI:** 10.1007/s11845-026-04381-4

**Published:** 2026-04-18

**Authors:** Fulden Sari, Zeliha Çelik, Deran Oskay, Nevin Aysel Güzel, Seriyye Allahverdiyeva, Halit Nahit Şendur, Şenol Çelik, Helene Alexanderson, Abdulsamet Erden, Abdurrahman Tufan

**Affiliations:** 1 Faculty of Physical Therapy and Rehabilitation, Department of Physiotherapy and Rehabilitation, Bingöl University, Bingöl, Türkiye; 2 Faculty of Health Science, Department of Physiotherapy and Rehabilitation, Amasya University, Amasya, Türkiye; 3 Faculty of Health Science, Department of Physiotherapy and Rehabilitation, Gazi University, Ankara, Türkiye; 4 Department of Radiology, Baku City Hospital, Baku, Azerbaijan; 5 Faculty of Medicine, Department of Radiology, Gazi University, Ankara, Türkiye; 6 Faculty of Agriculture, Department of Biometry and Genetics, Bingöl University, Bingöl, Türkiye; 7 Medical Unit Allied Health Professionals, Karolinska University Hospital, Theme Women’s Health and Allied Health Professionals, Stockholm, Sweden; 8 Department of Medicine, Division of Rheumatology, Karolinska Institutet, Solna, Stockholm, Sweden; 9 Faculty of Medicine, Department of Internal Medicine, Division of Rheumatology, Gazi University, Ankara, Türkiye

**Keywords:** Diaphragm, Diaphragm thickness, Inspiratory muscle, Lung, Respiratory function tests

## Abstract

**Background:**

Respiratory complications significantly result in morbidity and mortality in patients with Systemic Lupus Erythematosus (SLE). However, the impact of SLE on respiratory muscle function, particularly diaphragm thickness and strength, has been the subject of limited investigation.

**Aims:**

The study aims to investigate the key physiological and respiratory parameters affecting diaphragm thickness in SLE patients and to assess its role as a predictive indicator of respiratory dysfunction.

**Methods:**

The cross-sectional study included 29 patients with SLE and 48 healthy individuals. Diaphragm muscle thickness in both the inspiration and expiration phases was evaluated by ultrasonography. Fatigue, self-reported physical activity, pulmonary function tests, and respiratory muscle strength were assessed in the study.

**Results:**

Diaphragm thickness during inspiration and expiration phases, along with maximal inspiratory pressure (MIP) values, fatigue, and physical activity levels, were significantly lower in SLE patients compared to healthy controls (*p* < 0.05). Diaphragm thickness during the inspiratory phase showed a strong association with key pulmonary function parameters, particularly the FEV₁/FVC% and FEV₁%, while during expiration, it was mainly influenced by the FEV₁/FVC% and FVC%. Maximal expiratory pressure had a greater impact on diaphragm thickness compared to MIP. In SLE patients, the optimal cut-off values for diaphragm thickness were 1.50 mm during inspiration and 1.075 mm during expiration.

**Conclusion:**

These findings provide valuable information on diaphragm functionality and establish a strong foundation for clinical applications, emphasizing the potential of predictive models in diagnosing and managing respiratory dysfunction.

**Supplementary Information:**

The online version contains supplementary material available at https://doi.org/10.1007/s11845-026-04381-4.

## Introduction

Systemic lupus erythematosus (SLE) is a complex, chronic autoimmune disease characterized by widespread inflammation and multi-organ involvement, including the musculoskeletal, cardiovascular, renal, neurological, and respiratory systems [1, 2]. Respiratory complications are among the most significant contributors to morbidity and mortality in SLE patients, with conditions such as interstitial lung disease, pleural effusions, pulmonary hypertension, and respiratory muscle weakness frequently observed.^1^ While these complications are well documented, the impact of SLE on respiratory muscle function, particularly diaphragm thickness and strength, remains an area of limited research [3, 4].

Emerging evidence suggests that diaphragm thickness may serve as a valuable predictor of impaired respiratory muscle function and overall disease severity in various clinical conditions. In diseases such as chronic obstructive pulmonary disease (COPD) and heart failure, reductions in diaphragm thickness have been strongly correlated with impaired pulmonary function, decreased respiratory muscle strength, reduced exercise capacity, and increased mortality [5, 6]. Studies indicate that diaphragm atrophy in these conditions results from chronic inflammation, oxidative stress, and mechanical disadvantage caused by persistent hyperinflation, all contributing to respiratory dysfunction and poor clinical outcomes [7]. Despite its crucial role in respiratory functions, the investigation of diaphragm thickness in SLE remains notably unexplored in the existing literature. The few existing studies suggest that SLE patients may experience diaphragm thinning, which could be linked to respiratory impairment [3, 4]. A previous study reported that diaphragm thickness was significantly reduced in SLE patients, highlighting a potential link between SLE-related inflammation and diaphragm atrophy [4]. This thinning may be driven by the chronic inflammatory burden characteristic of SLE, leading to oxidative stress, muscle remodeling, and functional decline. Furthermore, the hyperinflation frequently observed in patients with compromised respiratory function could exacerbate diaphragmatic mechanical disadvantage, further impairing respiratory efficiency. Given these findings, investigating the role of diaphragm thickness on respiratory dysfunction in SLE could provide novel insights into disease-related respiratory dysfunction and its broader clinical implications [3, 4].

Although the aforementioned findings provide preliminary evidence for diaphragm involvement in SLE, there remains a notable gap in the literature regarding the specific predictors of diaphragm thickness and its clinical significance in this patient population. Therefore, this study aims to identify the key physiological and respiratory parameters influencing diaphragm thickness in SLE and to evaluate its potential as a predictive factor for respiratory dysfunction. By identifying key determinants of diaphragm thickness and elucidating its clinical relevance, this study aims to contribute to a deeper understanding of respiratory dysfunction in SLE and provide a foundation for improved diagnostic and therapeutic strategies.

## Method

### Study design

Approval for the cross-sectional study was obtained from the local ethics committee (Approval Date: 13/02/2024, Reference No: 24/3). Informed consent was attained from each participant prior to the evaluations, with patients reading and signing consent forms to confirm their voluntary participation. All study procedures adhered to the ethical principles established by the Declaration of Helsinki.

## Patients

The study included patients diagnosed with SLE according to the diagnostic criteria established by the European League Against Rheumatism (EULAR) and the American College of Rheumatology (ACR) [8], who were under regular follow-up at a tertiary Rheumatology center. Inclusion criteria for patients included: (1) age between 18 and 65 years; (2) being on a stable medical therapy; (3) stable general health for the previous 6 months; and (4) provision of informed and voluntary consent. Exclusion criteria for patients comprised: (1) inability to cooperate with assessments; (2) pregnancy; (3) any lung disease, including COPD, asthma, pneumonia, or interstitial lung disease (4) being on dialysis; (5) prior participation in a pulmonary rehabilitation program. All healthy individuals aged 18–65 years showed no signs of pulmonary disease (e.g., COPD, asthma) and had no known metabolic or endocrine disorders. These participants were enrolled from faculty staff and their healthy relatives who voluntarily agreed to participate. The patients were matched with healthy participants based on the statistical similarity of their age and gender. A flowchart presents the details of both included and excluded participants (Supplementary Fig. 1).

Patients with stable medical conditions were evaluated by a rheumatologist and subsequently referred to physiotherapy and rehabilitation services. Diaphragm thickness was measured by a radiologist specializing in this assessment technique. Further evaluations were conducted by a physiotherapist with expertise in rheumatic diseases.

### Assessments

Demographic information (gender, age, body mass index (BMI), smoking exposure), disease-specific details (disease duration, organ involvement), and current treatment were documented for all SLE patients on the day of their initial assessment. Disease activity was assessed using the SLEDAI-2 K, where higher scores indicate increased levels of disease activity [9].

**Fig. 1:**
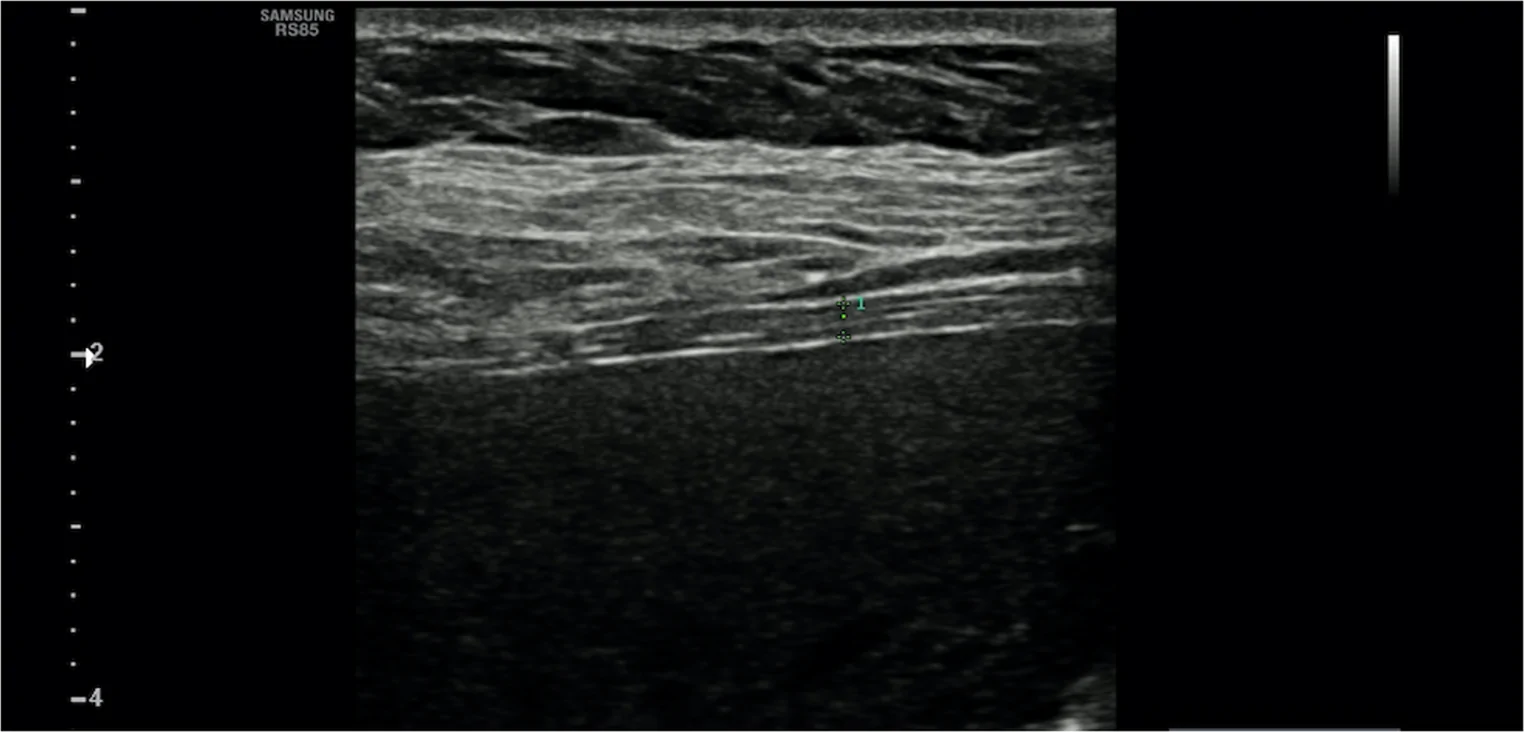
The evaluation of the thickness of the diaphragm (ultrasonography image)

*The diaphragm thickness* was evaluated by a single ultrasound device (RS 85, Samsung) with a linear transducer (Fig. 1). Measurements were taken after all participants were positioned supine with their hands on their heads. Assessments of diaphragm thickness were performed in the ninth or tenth intercostal space, mid-axillary level, with the probe held perpendicular to the chest wall. Three measurements were applied while in the breast-hold position of both the inspiration and expiration phases. The median value of assessments was recorded for analyses.

*The pulmonary functions* including Forced Vital Capacity (FVC), Forced Expiratory Volume in First Second (FEV_1_), FEV_1_/FVC, Peak Expiratory Flow (PEF), Forced Expiratory Flow from 25% to 75% (FEF_25–75%_) and *respiratory muscle strength*, including maximal inspiratory pressure (MIP) and maximal expiratory pressure (MEP), were measured using a spirometer (Pony FX, COSMED Inc., Italy). The procedures adhered to the guidelines set by the American Thoracic Society/European Respiratory Society on respiratory muscle and pulmonary function testing [10]. The pulmonary function test was performed 3 times, and the highest value was selected for analysis. For MIP, after a full expiration, the nasal was sealed with a nasal clip, and participants were instructed to perform a maximal inspiratory effort against a closed airway, maintaining the effort for 1–3 s. A similar method was applied for MEP measurements, where participants performed a maximal expiration against a closed airway following a full inspiration, also held for 1–3 s. The highest value was recorded. Higher MIP and MEP values indicate greater respiratory muscle strength [11, 12].

*The Fatigue Severity Scale (FSS)* consists of nine items, each rated on a scale from 1 (strongly disagree) to 7 (strongly agree). The total score is the mean of the nine items, with higher scores indicating greater fatigue severity [13].

*Physical activity levels* were assessed using the short form of the IPAQ-SF. This questionnaire estimates the total energy expenditure over the previous week, expressed in metabolic equivalent minutes per week (MET-min/week). The calculation involves evaluating the duration (minutes) and frequency (days) of various physical activities, including walking, moderate-intensity, and vigorous-intensity exercises. The total weekly physical activity score is determined by summing the MET-min/week values for each activity intensity category [14].

### Statistical analysis

The statistical analysis for this study was performed using IBM SPSS Statistics, version 26 (IBM Corp., 2019). To evaluate the normality of data distribution, both visual methods (such as probability plots and histograms) and analytical tests (Kolmogorov-Smirnov and Shapiro-Wilk) were applied. Continuous variables were presented as either mean ± standard deviation (SD) or median with interquartile range, depending on the data characteristics, while categorical variables were expressed as frequencies and percentages. For the comparison of continuous variables, either the student’s t-test or the Mann-Whitney U test was used based on whether the data followed a normal distribution. Statistical significance was defined as *p* < 0.05 for all analyses.

To assess the predictive performance of Chi-squared automatic interaction detection (CHAID), Classification and Regression Tree (CART), Automatic Linear Modeling (ALM), and Artificial neural networks (ANN) models, multiple goodness-of-fit metrics were calculated, including the Pearson correlation coefficient (r), Akaike Information Criterion (AIC), root-mean-square error (RMSE), coefficient of determination (R²), and adjusted R^2^. These metrics provided a comprehensive evaluation of model accuracy, allowing for comparisons across ten-fold cross-validations. CHAID and CART decision tree algorithms were employed to determine the most significant predictors of diaphragm thickness, while ALM was used to optimize linear modeling by automatically selecting the best subset of predictors. ANN, a machine-learning-based approach, was applied to capture complex, nonlinear relationships in the dataset. Machine learning algorithms, particularly CHAID, Exhaustive CHAID, and CART, have demonstrated superior predictive performance compared to traditional regression methods in multiple analytical studies. These approaches exhibit enhanced robustness to common statistical challenges such as multicollinearity and heteroscedasticity, which frequently compromise regression models. Machine learning techniques consistently achieve more favorable model performance metrics, including higher explained variance (R² and adjusted R²) and lower prediction errors (RMSE, MAPE, MAE), suggesting their utility in contexts requiring precise predictive modeling.

The ROC curve analysis was conducted to evaluate the diagnostic utility of diaphragm thickness in distinguishing between patients and healthy groups. Sensitivity (true positive rate) and 1-specificity (false positive rate) were plotted across different cut-off values to construct the ROC curve, with the area under the curve (AUC) serving as an indicator of classification performance. AUC values closer to 1.0 reflected superior discriminative power. Additionally, likelihood ratios and model performance trends were analyzed to refine the selection of optimal predictive models [15].

A post-hoc power analysis was carried out using G*Power software (version 3.1.9.2; Franz Faul, University of Kiel, Kiel, Germany). Diaphragm thickness during inspiration was chosen as the parameter for post-hoc evaluation. The analysis confirmed adequate statistical power (1-β > 95%, α = 0.05, d = 1.24, Df = 75).

## Results

Twenty-nine patients with SLE and 48 healthy individuals were included in the study. MIP, diaphragm thicknesses measured during inspiration and expiration, fatigue, and physical activity levels were significantly lower in the patients with SLE compared with the healthy individuals (Table 1; *p* < 0.05). The detailed demographic, clinical characteristics, and outcome measures of patients with SLE are presented in Table 1.

**Table 1:** Comparison of demographic characteristics, diaphragm structure and pulmonary functions in patients with SLE and healthy individuals

Characteristics	SLE (*n* = 29) (X ± SD) Median (IQR)	Health Individuals (*n* = 48) (X ± SD) Median (IQR)	*p*
Demographic Characteristics			
Age, year	33.03 ± 8.62	28.33 ± 11.48	0.061^*^
Female; Male, n/%	28/96.6%;1/3.4%	39/81.2%;9/18.8%	0.080
Weight, kg	66.41 ± 15.38	63.25 ± 12.77	0.333^*^
Height, m	1.64 ± 0.07	1.66 ± 0.09	0.304^*^
BMI, kg/m^2^	24.81 ± 5.30	23.02 ± 3.92	0.094^*^
Smoking exposure, pack*year	1.80 ± 3.73	1.19 ± 4.37	0.531^*^
Diaphragm thickness			
Inspiration, mm	0.99 ± 0.97	1.95 ± 0.50	**< 0.001** ^*^
Expiration, mm	0.60 ± 0.62	1.48 ± 0.40	**< 0.001** ^*^
Pulmonary parameters			
FVC, %	100.96 ± 11.96	99.70 ± 10.08	0.630^*^
FEV_1_%	96.30 ± 14.34	98.66 ± 10.32	0.415^*^
FEV_1_/FVC, %	95.69 ± 11.73	99.21 ± 7.99	0.130^*^
PEF, %	92.00 ± 21.87	89.74 ± 13.28	0.584^*^
FEF_2575_, %	84.19 ± 24.20	86.55 ± 20.87	0.659^*^
MIP, cmH_2_O	86.41 ± 22.96	104.10 ± 26.40	**0.004** ^*^
MEP, cmH_2_O	99.35 ± 35.63	110.13 ± 41.09	0.245^*^
Other parameters			
FSS total score	41.41 ± 13.00	33.10 ± 12.99	**0.011** ^*^
IPAQ-SF total score	360.0 (33.0–973.0.0.0)	1506.0 (876.25–4937)	**< 0.001** ^ᵠ^
SLEDAI-2 K score	5.81 ± 5.06		
Disease duration, year	10.42 ± 5.84		
Affected system			
Renal, %	44.7%		
Hematological, %	3.4%		
Neurological, %	6.8%		
Active ingredient of the medicine			
Hydroxychloroquine sulfate	86.2%		
Mycophenolate mofetil	31.03%		
Prednisolone	41.37%		

*FEV*_*1*_, Forced expiratory volume in the first second; *FVC*, Forced vital capacity; *FEV*_*1*_*/FVC*, Forced expiratory volume in the first second/forced vital capacity; *PEF*, Peak expiratory flow; *FEF*_*25–75%*_, Forced expiratory flow from 25% to 75%; *MIP*, Maximal inspiratory pressure; *MEP*, Maximal expiratory pressure; *cmH*_*2*_*O*, centimeter water; mm, millimeter; IPAQ-SF, International Physical Activity Questionnaire-Short Form; SLEDAI-2 K, Systemic Lupus Erythematosus Disease Activity Index 2000. SD: Standard deviation, IQR25th-75th: interquartile range 25–75. *: Independent-samples T-Test, ᵠ: Mann–Whitney U Test. *p* < 0.05

**Fig. 2:**
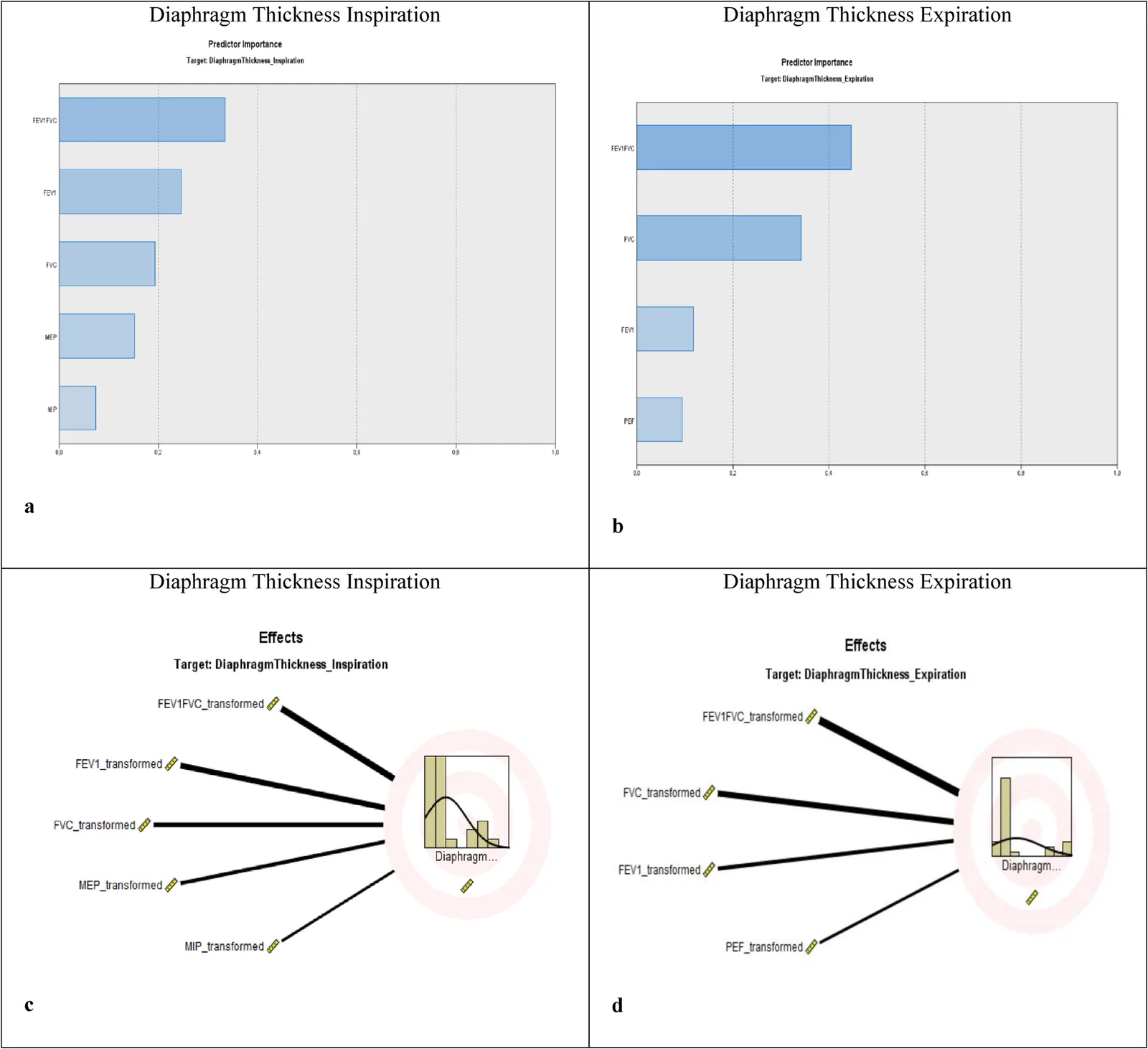
Importance levels of the variables or predictors

The importance levels of predictors influencing diaphragm thickness during both the inspiration and expiration phases were analyzed using a linear modeling approach. For inspiration, FEV_1_/FVC% emerged as the most critical variable, contributing 33.5% of the model’s importance, followed by FEV_1_%, FVC%, MEP, and MIP (Fig. 2a). Additionally, for expiration, FEV_1_/FVC%, FVC%, FEV_1_%, and PEF% were the most significant contributors (Fig. 2b). The ANOVA results for the multiple linear regression analysis are presented in Fig. 2c and d. In these figures, the thickness of each line reflects the statistical significance of the associated predictor, while the predictors are arranged from top to bottom according to their relative importance. These current findings underline the vital role of pulmonary function parameters in determining diaphragm thickness, emphasizing the physiological connection between respiratory capacity and diaphragm functionality. The ALM analysis assessed the predictability of the mean diaphragm thickness inspiration and expiration scores, identifying the most influential morphological characteristics and their respective significance values, as presented in Table 2.

**Table 2:** Coefficients determined for the diaphragm thickness inspiration and expiration target variable

Model Term	Coefficient	*p*-Value	Importance
Diaphragm Thickness Inspiration			
Intercept	−5.050	0.011	
FEV_1_/FVC%	0.071	0.001	0.335
FEV_1_	−0.079	0.002	0.246
FVC	0.074	0.005	0.194
MEP	−0.014	0.012	0.152
MIP	0.016	0.070	0.074
Diaphragm Thickness Expiration			
Intercept	−3.993	0.004	
FEV_1_/FVC%	0.051	0.000	0.446
FVC	0.055	0.001	0.342
FEV_1_	−0.042	0.041	0.117
PEF	−0.013	0.065	0.094

*p* < 0.05. *FEV*_*1*_, Forced expiratory volume in the first second; *FVC*, Forced vital capacity; *FEV*_*1*_*/FVC*, Forced expiratory volume in the first second/forced vital capacity; *PEF*, Peak expiratory flow *MIP*, Maximal inspiratory pressure; *MEP*, Maximal expiratory pressure

The predictive analysis of diaphragm thickness during both the inspiration and expiration phases demonstrates the efficacy of different modeling approaches. For inspiration, ANN exhibited the highest predictive accuracy, achieving the strongest correlation (*r* = 0.900), the highest coefficient of determination (R²=0.810), and the lowest root mean square error (RMSE = 0.363), establishing ANN as the most reliable model. Conversely, for expiration, the CHAID model outperformed others with the highest adjusted R² (0.756) and the lowest RMSE (0.378), while the CART algorithm demonstrated the weakest performance (Table 3). These results suggest that ANN is the most effective model for diaphragm thickness prediction during inspiration, whereas CHAID provides the best predictive accuracy for expiration measurements.

The predictive analysis demonstrates that MLP ANN performs best for diaphragm thickness inspiration and expiration, influencing sensitivity and specificity. Additionally, ANN was found to be the most effective method for predicting diaphragm thickness across both phases, while ALM provided reliable alternative approaches. (Supplementary Fig. 2). The study highlights the critical influence of “FEV1/FVC%” and other respiratory function parameters, providing a robust foundation for clinical applications. These findings not only enhance the understanding of diaphragm functionality but also offer a robust foundation for clinical applications, reinforcing the potential of predictive modeling in diagnosing and managing respiratory diseases.

**Table 3:** CHAID, CART, ALM, and ANN types’ predictive performance

	Diaphragm Thickness Inspiration				Diaphragm Thickness Expiration			
	CHAID	CART	ALM	ANN	CHAID	CART	ALM	ANN
r	0.828	0.755	0.856	0.900	0.875	0,780	0,885	0,865
R^2^	0.686	0.570	0.733	0.810	0.766	0,624	0,783	0,748
Adjusted R^2^	0.673	0.561	0.710	0.780	0.756	0.616	0,761	0.722
RMSE	0.508	0.594	0.499	0.363	0.378	0.647	0.415	0.391
AIC	−67.445	−52.148	−65.378	−94.594	−98.278	−43.208	−86.454	−86.563

CHAID, Chi-Squared Automatic Interaction Detection; CART, Classification and Regression Tree; ALM, Automatic Linear Modeling; ANN, Artificial Neural Networks; AIC, Akaike Information Criterion; RMSE, Root Mean Square Error; R², Coefficient of Determination

**Fig. 3:**
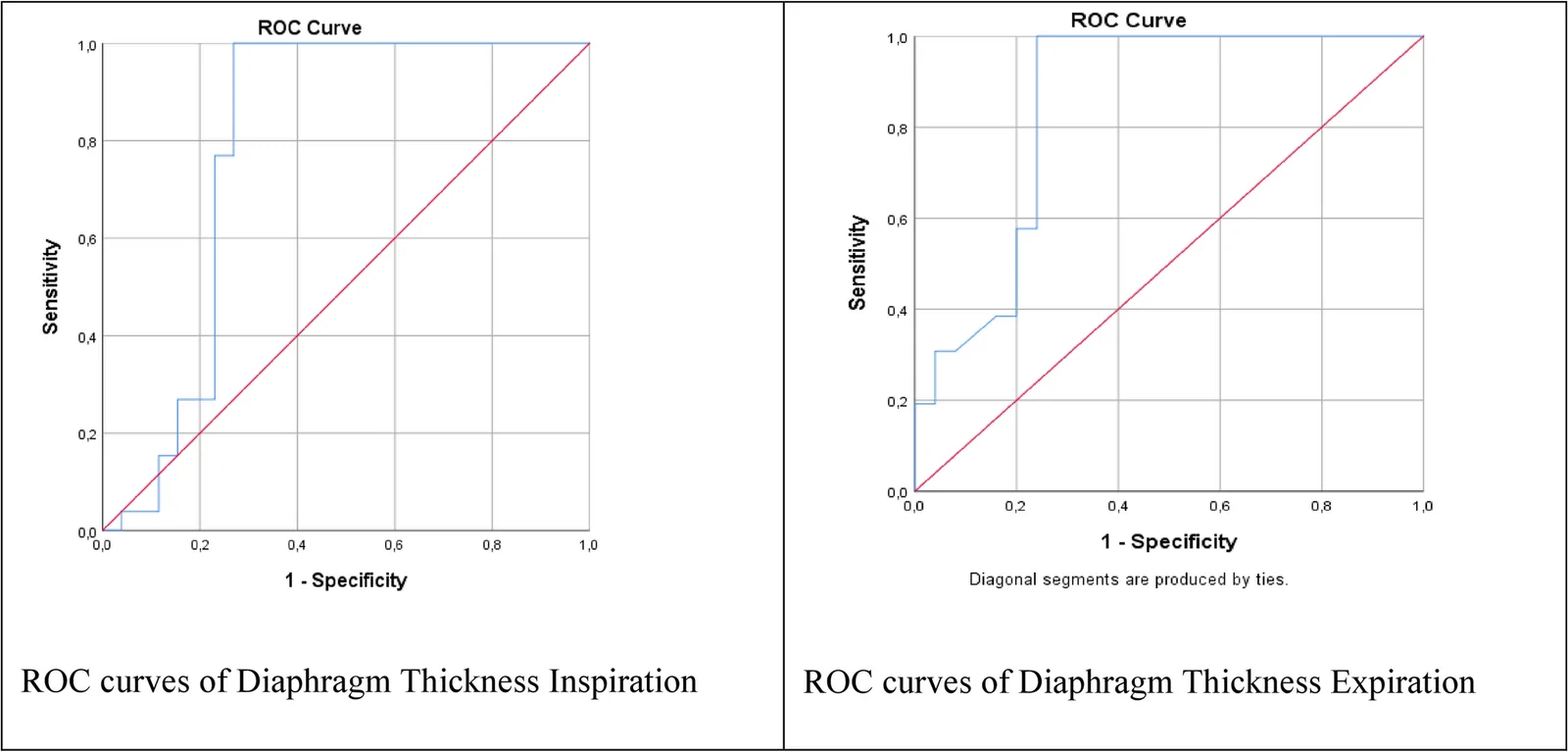
Roc curve of diaphragmatic thickness inspiration and expiration

In the study, the predictive power of diaphragm thickness during both inspiration and expiration phases in distinguishing patients from healthy individuals was evaluated using ROC analysis. The area under the curve (AUC) for diaphragm thickness during inspiration was determined as 0.790 (95% CI: 0.646–0.934, *p* < 0.001), with an optimal cut-off point of 1.50. Similarly, the AUC for diaphragm thickness during expiration was calculated as 0.846 (95% CI:0.729–0.963, *p* < 0.001), with an optimal cut-off point of 1.075 (Fig. 3). The current findings suggest that diaphragm thickness in both inspiration and expiration phases serve as strong predictors for differentiating patients from healthy individuals.

## Discussion

The primary findings of the study indicate that diaphragm thickness during the inspiratory phase exhibits a strong association, particularly with the FEV₁/FVC% and FEV_1_%, which are key pulmonary function parameters. In contrast, during the expiratory phase, the most significant influences on diaphragm thickness were observed to be the FEV₁/FVC% and FVC%. Diaphragm thickness is a dynamic parameter influenced by complex respiratory interactions as well as physiological and mechanical factors. From the perspective of respiratory system function, these factors represent a critical area of research concerning diaphragm thickness regulation. Another key factor is that MEP was found to have a more pronounced effect on diaphragm thickness than MIP. This observation can be explained by multiple physiological and mechanical mechanisms. Additionally, in patients with SLE, the optimal cut-off values for diaphragm thickness during inspiration and expiration were determined to be 1.50 mm and 1.075 mm, respectively. Furthermore, both inspiratory and expiratory diaphragm thickness, as well as MIP values, were found to be significantly lower in SLE patients compared to healthy individuals.

The diaphragm plays a critical role in respiratory function, and its structural integrity is essential for maintaining adequate ventilation and respiratory muscle performance [16]. In this study, we demonstrated that the diaphragm thickness in our healthy group was consistent with the baseline values reported in the literature for healthy individuals. Specifically, Carrillo-Esper et al. reported standardized values for diaphragm thickness at rest in younger healthy adults, with a mean inspiratory diaphragm thickness of 1.9 mm and a 95% confidence interval of 1.7–2.0 mm [17], further supporting the reliability of our healthy control measurements. When compared to healthy controls, patients with SLE in our study exhibited a significant reduction in diaphragm thickness during both the inspiration and expiration phases. This finding aligns with previous studies indicating that chronic inflammatory diseases, such as SLE, can lead to structural and functional impairments in respiratory muscles [7, 18, 19]. Similar observations have been made in other chronic diseases; Levine et al. showed that the cross-sectional area of diaphragm fibers was reduced in patients with advanced COPD [18], while Ottenheijm et al. reported that patients with mild to moderate COPD had similar diaphragm fiber areas compared to healthy controls, emphasizing diaphragm dysfunction may vary depending on the severity of the disease [19]. To our knowledge, a few studies have specifically investigated diaphragm function in SLE patients [3, 4]. One study by Satis et al. compared SLE patients with those with Sjögren’s syndrome but did not include a healthy control group [4]. The second study was a case-control study that compared diaphragm thickness before and after IMT treatment [3]. Our study is unique in demonstrating a significant reduction in diaphragm thickness through direct comparison with healthy controls, offering a valuable contribution to the limited body of literature on diaphragm involvement in SLE.

In our study, the patients did not present with either pulmonary symptoms or a clinically diagnosed pulmonary involvement. However, despite the absence of overt pulmonary disease, the diaphragm thickness during both inspiration and expiration was significantly reduced compared to healthy controls. In addition, the MIP values in SLE patients were also found to be lower than those of the healthy group. These findings align with previous studies conducted on SLE patients, which have similarly reported significant reductions in MIP values compared to healthy individuals [4, 20] Jacobelli et al. highlighted that in the absence of pulmonary involvement, inspiratory muscle dysfunction may be a cause of reduced lung volumes commonly observed in SLE patients. Moreover, their study stated that the range of abnormalities can vary from mild inspiratory muscle dysfunction to more severe impairments, potentially leading to conditions such as shrink lung syndrome and unexplained dyspnea [20]. In addition to the findings of Jacobelli et al., our study suggests that the reduction in diaphragm thickness, even in the absence of clinically evident pulmonary involvement, could potentially contribute to the development of various complications and poor prognosis in the long term. Furthermore, consistent with the literature, our study demonstrated that fatigue and physical activity levels in SLE patients were significantly lower than those observed in the healthy control group [2, 21].

Recent studies have increasingly indicated that diaphragm thickness is significantly reduced in patients with chronic conditions such as COPD, heart failure, and systemic sclerosis [5, 6, 22–24]. A study has shown the inverse relationship between diaphragm muscle thickness and overall health status, with particular emphasis on chronic respiratory conditions [22]. Cimsit et al. found that diaphragm thickness in mild COPD patients was primarily related to FEV_1_%, highlighting the importance of lung function as a potential predictor in the early stages of the disease [25]. In our study, based on advanced statistical results from the applied ALM analysis, we found that inspiratory diaphragm thickness was primarily influenced by FEV_1_/FVC% (33.5%) and FEV_1_% (24.6%), while expiratory diaphragm thickness was predominantly affected by FEV_1_/FVC% (44.6%) and FVC% (34.2%). Despite the relationship between reduced diaphragm thickness and chronic diseases, a vital limitation in recent studies is the insufficient exploration of key predictive factors contributing to diaphragm atrophy. This gap causes a significant challenge to the development of targeted therapeutic strategies designed to prevent or mitigate diaphragm atrophy, underscoring the need for more comprehensive investigations into the underlying mechanisms and risk factors. To the best of our knowledge, our study is not only the first to identify the predictors influencing diaphragm thickness in patients with SLE but also highlights significant findings aimed at addressing this gap in the literature.

According to the modeling results in our study, one of the most significant findings is that MEP is a stronger predictor of diaphragm thickness compared to MIP. One possible explanation is that activation of abdominal and accessory muscles during expiration increases intra-abdominal pressure, influencing diaphragm position and contraction dynamics. Therefore, intra-abdominal pressure changes during expiration may play a role in determining diaphragm thickness during inspiration [26, 27]. Another potential explanation relates to the structural composition and physiological adaptations of the diaphragm muscle. The diaphragm is composed of both slow-twitch (type I) and fast-twitch (type II) muscle fibers, with the proportion of these fibers varying among individuals [27, 28]. The diaphragm’s physiological response to pressure changes during the expiratory phase may be influenced by its muscle fiber composition, potentially enhancing the effect of MEP on diaphragm thickness during inspiration. We may suggest that this finding from our study will provide valuable insights for future studies.

ROC analysis plays a crucial role in identifying diaphragm thickness as a reliable predictor of respiratory function. The proposed cut-off values may assist in the early detection of respiratory dysfunction and support treatment planning in patients with SLE [29, 30]. Kareem et al. stated that diaphragmatic ultrasonographic thickness demonstrated limited diagnostic utility in COPD, possibly due to the minimal nature of the observed changes or the overlap of these changes with those found in various respiratory disorders [29]. Another study, defined diaphragmatic atrophy using a cut-off value of less than 2 mm for diaphragm thickness measured at the end of expiration [30]. In the present study, the determined cut-off values for diaphragm thickness during inspiration and expiration in SLE patients were 1.50 mm and 1.075 mm, respectively. The study is the first to establish and highlight the cut-off values for diaphragm thickness during inspiration and expiration in patients with SLE, marking a significant contribution to the field of respiratory assessment in autoimmune diseases.

The study has some limitations. Firstly, the SLE group included only one male participant. As Carrillo-Esper et al. [16] highlighted, significant differences in diaphragm thickness exist between genders, suggesting that future studies should consider separate analyses by sex. Secondly, diaphragm mobility and elastography were not evaluated, representing another limitation of this study. However, the most significant strength of our study lies in its originality, as it is the first to demonstrate the predictive importance of diaphragm thickness during inspiration and expiration in connective tissue diseases, expanding on earlier research involving SLE patients.

In conclusion, the study highlights the importance of diaphragm thickness as a valuable indicator of respiratory function parameters (FEV₁/FVC%, FEV₁%, and FVC%) in SLE patients, with MEP emerging as a key influencing factor. Establishing diaphragm thickness as a predictive factor in SLE could facilitate earlier detection of respiratory involvement, optimize disease monitoring, and inform targeted interventions to enhance patient outcomes. The cut-off values provided could serve as a reference for future clinical assessments of respiratory dysfunction in connective tissue diseases.

## Supplementary Information

Below is the link to the electronic supplementary material.

Supplementary Material 1

Supplementary Material 2

## Data Availability

The data that support the findings of this study are available from the principal investigator (Fulden Sari) upon reasonable request and with appropriate ethical oversight.
