# Supplementary material for: Ultrasonographic assessment of the diaphragm in systemic lupus erythematosus: cut-off values and predictive indicators of diaphragm thickness
Source: Ir J Med Sci. 2026 Apr 18;195(4):2221–30. doi: 10.1007/s11845-026-04381-4 (PMC13588865; doi:10.1007/s11845-026-04381-4)
Supplement: Supplementary file 1 [file 11845_2026_4381_MOESM1_ESM.docx]

SLE Group	Healthy Group

(n=37)	(n=53)

Exlusion (n=8)	Exlusion (n=5)

Having femoral impingement syndrome (n=1)	Doing regular exercise (n=2)

Not meeting the age requirement (n=3)	Unwillingness to participate (n=3)

Unwillingness to participate (n=3)

Active respiratory tract infection (n=1)

Analyzed (n=29)	Analyzed (n=48)

**Supplementary 1.** Flow chart of the participants
