# Supplementary material for: Ultrasonographic assessment of the diaphragm in systemic lupus erythematosus: cut-off values and predictive indicators of diaphragm thickness
Source: Ir J Med Sci. 2026 Apr 18;195(4):2221–30. doi: 10.1007/s11845-026-04381-4 (PMC13588865; doi:10.1007/s11845-026-04381-4)
Supplement: Supplementary file 2 [file 11845_2026_4381_MOESM2_ESM.docx]

**Supplementary 2a.** Structure of the MLP ANN of Diaphragm Thickness Inspiration

**Supplementary 2b.** Structure of the MLP ANN of Diaphragm Thickness Expiration
